# The Anti-ADAMTS-5 Nanobody^®^ M6495 Protects Cartilage Degradation Ex Vivo

**DOI:** 10.3390/ijms21175992

**Published:** 2020-08-20

**Authors:** Anne Sofie Siebuhr, Daniela Werkmann, Anne-C. Bay-Jensen, Christian S. Thudium, Morten Asser Karsdal, Benedikte Serruys, Christoph Ladel, Martin Michaelis, Sven Lindemann

**Affiliations:** 1 ImmunoScience, Nordic Bioscience Biomarkers and Research, 2730 Herlev, Denmark; acbj@nordicbio.com (A.-C.B.-J.); cst@nordicbio.com (C.S.T.); MK@nordicbio.com (M.A.K.); 2 Merck KGaA, 64293 Darmstadt, Germany; daniela.werkmann@merckgroup.com (D.W.); christoph.ladel@merckgroup.com (C.L.); martin.michaelis@merckgroup.com (M.M.); sven-lindemann@merckgroup.com (S.L.); 3 Ablynx, A Sanofi Company, 9052 Ghent, Belgium; benedikte.serruys@ablynx.com

**Keywords:** ADAMTS-5, aggrecan, cartilage, biomarkers

## Abstract

Osteoarthritis (OA) is associated with cartilage breakdown, brought about by ADAMTS-5 mediated aggrecan degradation followed by MMP-derived aggrecan and type II collagen degradation. We investigated a novel anti-ADAMTS-5 inhibiting Nanobody^®^ (M6495) on cartilage turnover ex vivo. Bovine cartilage (BEX, *n* = 4), human osteoarthritic - (HEX, *n* = 8) and healthy—cartilage (hHEX, *n* = 1) explants and bovine synovium and cartilage were cultured up to 21 days in medium alone (*w*/*o*), with pro-inflammatory cytokines (oncostatin M (10 ng/mL) + TNFα (20 ng/mL) (O + T), IL-1α (10 ng/mL) or oncostatin M (50 ng/mL) + IL-1β (10 ng/mL)) with or without M6495 (1000−0.46 nM). Cartilage turnover was assessed in conditioned medium by GAG (glycosaminoglycan) and biomarkers of ADAMTS-5 driven aggrecan degradation (huARGS and exAGNxI) and type II collagen degradation (C2M) and formation (PRO-C2). HuARGS, exAGNxI and GAG peaked within the first culture week in pro-inflammatory stimulated explants. C2M peaked from day 14 by O + T and day 21 in co-culture experiments. M6495 dose dependently decreased huARGS, exAGNxI and GAG after pro-inflammatory stimulation. In HEX C2M was dose-dependently reduced by M6495. M6495 showed no effect on PRO-C2. M6495 showed cartilage protective effects by dose-dependently inhibiting ADAMTS-5 mediated cartilage degradation and inhibiting overall cartilage deterioration in ex vivo cartilage cultures.

## 1. Introduction

The extracellular matrix (ECM) of cartilage is well described and has been studied for decades. It is recognized that not only cartilage, but several joint tissues are important in osteoarthritis (OA) pathology. However, cartilage is still the main focus area in development of disease modifying treatments for OA (DMOADs). The ECM of cartilage consists mainly of proteoglycans and collagens and the chondrocyte is the only cell type responsible for the maintenance of cartilage. With the few ECM molecules and the single cell type present in cartilage, the search for DMOADs should be simple, but the continuous failure of DMOADs in phase 2 and 3 trials tells otherwise.

ECM turnover is dependent on a delicate regulation of synthesis and degradation during development and aging. During aging or arthritic diseases, the proteolytic degradation of cartilage ECM is increased compared to the formation. The main ECM proteins in cartilage are aggrecan and type II collagen. ADAMTS-4 (aggrecanase-1) and ADAMTS-5 (aggrecanase-2) have been identified as key enzymes for proteolytic depletion of aggrecan during the development of OA [1,2]. Both ADAMTS-4 and ADAMTS-5 cleave the NITEGE373↓374ARGS bond in the interglobular domain (IGD) of aggrecan. MMP-13 has been recognized as the key protease for initial collagen type II degradation [3]. Previous studies in mice have indicated that while MMP-mediated cartilage destruction leads to irreversible deterioration of the joint matrix, the aggrecanase-mediated cartilage deterioration was fully reversible upon correction of the catabolic environment [4,5,6]. Other in vivo studies have found that aggrecan loss was reversible as long as the progression was not too advance [7,8,9]. In a murine model inhibition of ADAMTS-5 prevented overall cartilage degradation, suggesting that ADAMTS-5 may be the primary aggrecanase responsible for aggrecan degradation in mice [10]. Inhibiting MMP-13, another protease upregulated during OA, reduced collagen degradation, but not aggrecan degradation [11]. These results indicate that inhibiting aggrecan degradation by targeting aggrecanases may be more chondroprotective than targeting collagen degradation. In addition, studies have demonstrated a superior effect of selective monoclonal antibody to ADAMTS-5 versus ADAMTS-4 in human OA cartilage explants [12]. Thus, ADAMTS-5 is an attractive target in the development of a therapeutic agent for OA [13].

In general, degradation of the aggrecan net results in the generation and release of glycosaminoglycan (GAG) fragments [14]. Specific cleavage with ADAMTS-5 in aggrecan exposes the ARGS neo-epitope; the N-terminal neo-epitope after cleavage of the NITEGE373↓374ARGSV bond in aggrecan [15] The ARGS fragment is rapidly excreted from the ECM in cartilage explant culture models and is also present in serum, urine and synovial fluid of arthritis patients [16,17]. Quantification of the ARGS fragment in synovial fluid, instead of the full length aggrecan, have been found to be a more sensitive biomarker to distinguish injured and diseased subjects from normal, than other aggrecanase-derived biomarkers [14,18].

In this study we investigated the inhibitory effect of the Nanobody^®^ M6495, specifically inhibiting ADAMTS-5, in ex vivo cartilage models. M6495 is a bivalent and bifunctional Nanobody of 28.1 kilodalton (kDa) comprising two sequence-optimized variable domains derived from heavy chain-only llama antibodies, i.e., one N-terminal ADAMTS-5-neutralizing building block and one C-terminal human serum albumin (HSA)-binding block for in vivo half-life extension (HLE) (See Figure S1). The two building blocks are separated by a glycine-serine linker (35GS).

## 2. Results

### 2.1. M6495 Specificity towards ADAMTS-5

**Figure 1:**
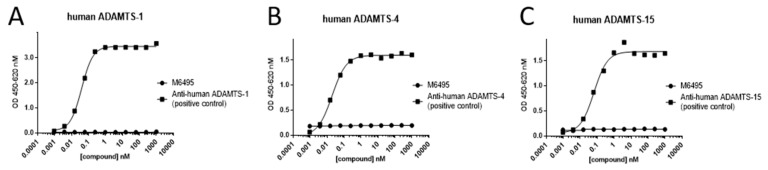
Specificity of the M6495 Nanobody. (**A**–**C**), M6495 do not bind to ADAMTS, 1, 4 and 15.

M6495 showed a high affinity for its target ADAMTS-5, with a KD of 3.65 pM (95% CI: 2.27 pM–5.86 pM) (*n* = 3, CV 20%). Also, M6495 demonstrated full functionality against its target, as indicated by a concentration-dependent and complete inhibition of the enzymatic activity of ADAMTS-5 by M6495 in an enzymatic activity assay (unpublished data). In contrast to its binding to ADAMTS-5, M6495 did not bind to either ADAMTS-1, ADAMTS-4 or ADAMTS-15 (Figure 1).

### 2.2. Metabolic Activity

The metabolic activity of untreated bovine and OA human cartilage explants was stable throughout the cultures, whereas the pro-inflammatory stimulations (O + T, IL-1α and IL-1β) lowered the metabolic activity by the end of the bovine and OA human cartilage studies (See Figure S2). With healthy human cartilage explants the metabolic activity dropped after day seven especially in the O + T treated explants.

### 2.3. ECM Turnover in Bovine and OA Human Cartilage

**Figure 2:**
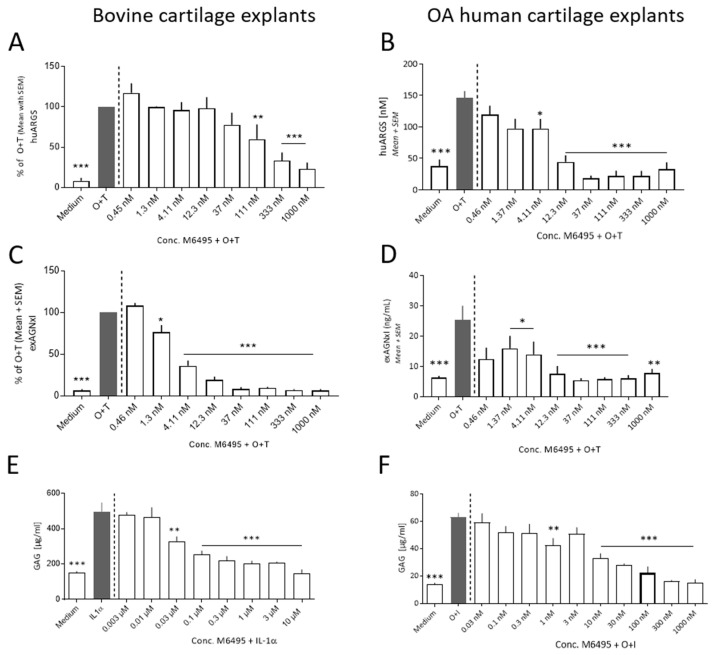
Aggrecan turnover in bovine cartilage explants and OA human cartilage explants at day 5 of culture. (**A**,**B**): ADAMTS-5 derived aggrecan degradation (huARGS). (**C**,**D**): MMP-derived aggrecan degradation (exAGNx1). (**E**,**F**): Glycosaminoglycan (GAG) release. The bovine cartilage explant data displayed for huARGS and AGNxI were from study 2 and GAG was from study 4. Statistical significance was tested by one-way ANOVA with Dunnett’s multiple comparisons test comparing to O + T, IL-1α or O + I. * *p* < 0.05; ** *p* < 0.01; *** *p* < 0.001.

Aggrecan degradation (huARGS, exAGNxI and GAG) was assessed at day five in the bovine and OA human cartilage explants. O + T induced a significant release of huARGS and exAGNxI compared to medium (*p* < 0.001; Figure 2A,D). The release peak of huARGS was at day five in all studies, whereas the exAGNxI peak release was at day five or seven depending on the experiment (Figures S3 and S4). The O + T induced release of huARGS and exAGNxI in bovine cartilage explants were dose-dependently inhibited by M6495 (Figure 2A,C). In OA human cartilage explants the O + T induced release of huARGS was also dose-dependently inhibited by M6495, whereas a general lowering with a tendency towards dose-dependent inhibition of exAGNxI with M6495 was observed (Figure 2B,D). Treatment with IL-1α significantly induced GAG release compared to medium. The IL-1α induced GAG release was dose-dependently inhibited by M6495 (Figure 2E,F).

**Figure 3:**
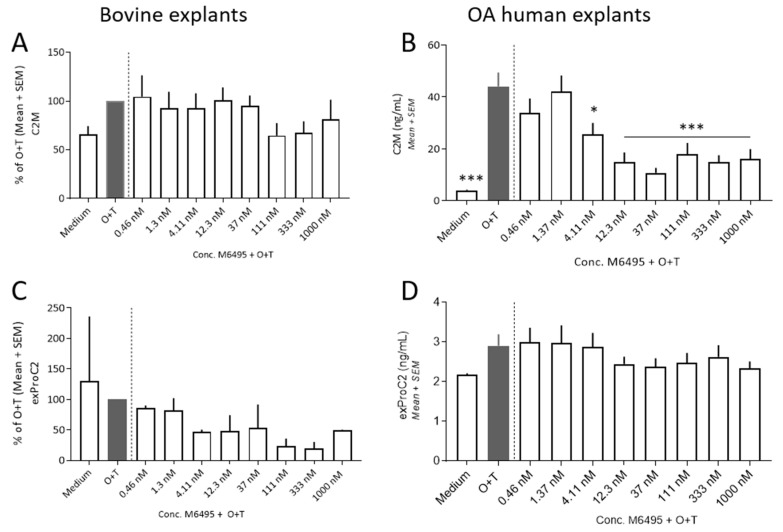
Cartilage turnover in bovine cartilage explants and OA human cartilage explants at day 14 of culture. (**A**,**B**): MMP-derived collagen type II degradation (C2M). (**C**,**D**): Formation of type II collagen (exPRO-C2). Statistical significance was tested by one-way ANOVA with Dunnett’s multiple comparisons test comparing to O + T.; * *p* < 0.05; *** *p* < 0.001.

The type II collagen turnover (C2M and exPRO-C2) was assessed in bovine cartilage and OA human cartilage explants. The C2M released was induced by O + T from day 14 and throughout the remainder of the culture period (Figure S3). The O + T induced release of C2M was inhibited by M6495 in OA human cartilage explants (Figure 3C), but not in the bovine cartilage explants (Figure 3A). O + T and O + T + M6495 did not affect the exPRO-C2 release at any time point compared to medium alone (Figure 3B,D).

### 2.4. ECM Turnover in Healthy Human Cartilage

**Figure 4:**
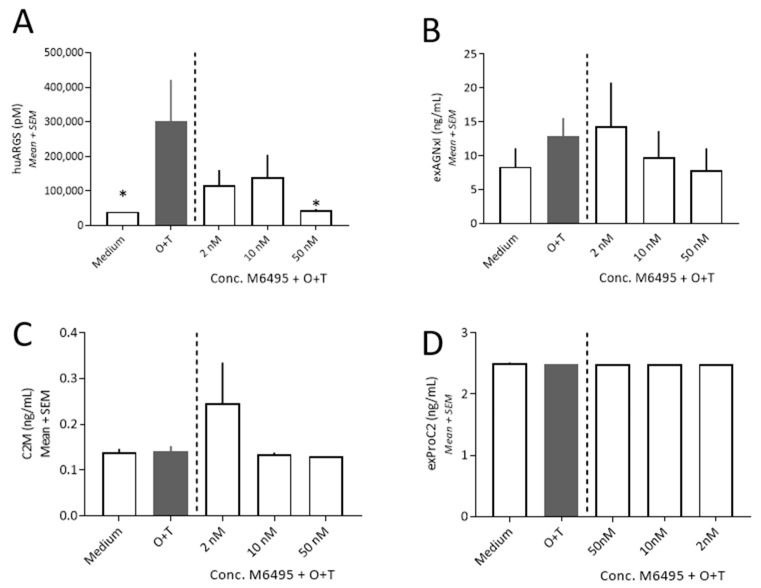
Cartilage turnover in healthy human cartilage explants. (**A**): ADAMTS-5 derived aggrecan degradation (huARGS) at day 5 of culture. (**B**): MMP-derived derived aggrecan degradation (exAGNx1) at day 5 of culture. (**C**): MMP-derived type II collagen (C2M) at day 21 of culture. (**D**): Formation of type II collagen (exPRO-C2) at day 21 of culture. Statistical significance was tested by one-way ANOVA with Dunnett’s multiple comparisons test comparing to O + T.; * *p* < 0.05.

O + T addition was used to stimulate ECM degradation in healthy human cartilage explants. The aggrecan degradation was assessed on day 5 as in the bovine and OA human cartilage explants. In the healthy human cartilage explants O + T significantly increased the release of huARGS (*p* = 0.028), while O + T did not significantly induce exAGNxI release compared to medium albeit an upwards trend was observed (Figure 4A,B). M6495 at 50 nM inhibited the O + T induced huARGS release in healthy human cartilage and showed a tendency towards a dose-dependent inhibition of exAGNxI. M6495 at 50 nM was at the level of negative control.

O + T did not induce a C2M release compared to medium at day 21 and M6495 did not alter the C2M release, albeit one explant of the O + T + M6495 (2 nM) had increased C2M (Figure 4B). O + T did also not induce exPRO-C2 in healthy human cartilage explants and M6495 did not affect the exPRO-C2 release (Figure 4D).

### 2.5. Co-Cultures

**Figure 5:**
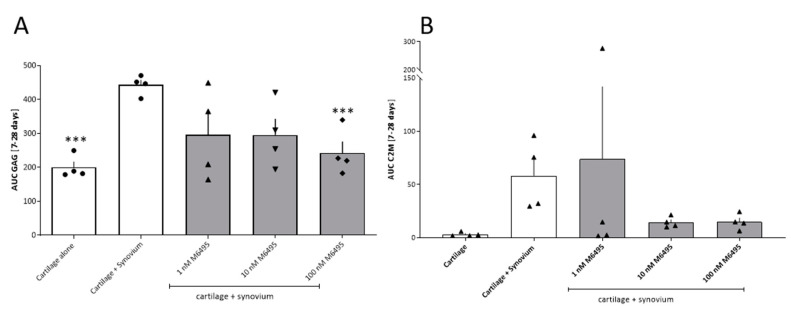
Cartilage turnover in bovine cartilage explants in co-culture with bovine synovial membrane. (**A**): The AUC of glycosaminoglycan (GAG) release during culture (day 7 to 28). (**B**): MMP-derived type II collagen degradation (C2M) during culture (day 7 to 28). Statistical significance was tested by one-way ANOVA with Dunnett’s multiple comparisons test comparing to cartilage + synovium.; *** *p* < 0.001.

A co-culture of bovine cartilage and synovium was done, where the pro-inflammatory induction was driven by the synovium instead of stimulation with exogenous cytokines. Aggrecan turnover in the bovine co-culture was assessed only with GAG release in the supernatant at day 7, 14, 21 and 28 (Figure S5). Addition of the synovium to the cartilage significantly increased the release of GAG over time with the peak GAG release observed at day 7 (Figure S5). M6495 (1, 10, 100 nM) dose-dependently inhibited the GAG release reaching statistical significance with 100 nM M6495 (Figure 5A). The C2M level started to increase after 21 days in the co-culture compared to cartilage explants alone (without reaching statistical significance). M6495 (1, 10, 100 nM) trended to inhibit the C2M release with one technical replicate reaching a higher C2M than O + T alone (Figure 5B).

## 3. Discussion

Halting cartilage degradation is still the major focus of DMOADs for OA. We investigated a potential DMOAD (a Nanobody, M6495) inhibiting ADAMTS-5 in cartilage ex vivo models to characterize its chondroprotective effects. Overall, we found that M6495 significantly inhibited pro-inflammatory cytokine induced cartilage degradation by assessment of aggrecan and type II collagen degradation. Furthermore, M6495 did not show any effect on type II collagen production. These results indicate M6495 as a potential anti-catabolic treatment for OA as it may halt cartilage degradation.

An efficient DMOAD should modify the underlying OA pathophysiology and thereby halt the structural damage to prevent or reduce long-term disability. Currently, no DMOAD is approved for OA, but several clinical trials are ongoing [19]. One group of promising DMOADs are the drugs targeting the proteolytic activity, such as ADAMTS-4 and -5, Cathepsin K, and MMPs. Another group of DMOADs targets growth factors and cytokines, such as FGF18 and TGF-beta and here some programs are in late stage development and especially FGF-18 (sprifermin) have shown promising results as an anabolic treatment for OA [20]. Drugs aimed at collagen degradation, such as MMP inhibitors, have been tested, but early drugs tested showed musculoskeletal syndrome side-effects with collagen build-up within other tissues [21,22]. Targeting aggrecan degradation may be a safer alternative.

A biomarker of aggrecanase degraded aggrecan, such as ARGS is expected to significantly accelerate the clinical development of aggrecanase inhibitors and other disease modifying drugs for OA [23,24]. Furthermore, such a biomarker has the potential to serve as an activity and prognostic biomarker to facilitate patient selection and be a pharmacodynamic biomarker for OA clinical trials. It was previously reported that serum ARGS levels in a moderate OA group were significantly higher compared to a non-OA control group. However, there was no difference in the urinary ARGS levels between the diseased and control groups [25]. Furthermore, the level of ARGS in synovial fluid and serum were elevated remarkably after anterior cruciate ligament injury in the knee [26]. Another study found significant decreases in synovial fluid ARGS concentrations from baseline to follow-up in patients with acute knee injury [27]. The level of synovial fluid ARGS have also been inversely associated with increased loss of joint space in patients with previous meniscectomy in the knee, but without radiographic knee OA [28].

Several ADAMTS-5 inhibitors have been developed (Agg523, GLPG1972, GSK2384002, CRB0017), but only a few are or have been developed further than phase 1 clinical trials. The Nanobody^®^ M6495 has been tested in healthy male subject in a Phase I study, where it showed acceptable safety and tolerability profile [29]. In addition, tested doses did not affect cardia conduction and no evidence of QT prolongation was observed [29]. Furthermore, another ADAMTS-5 inhibitor, GLPG1972, was also found to have an acceptable safety and tolerability profile in a Phase I study [30]. The AGG523 had poor pharmacokinetics and was terminated after phase 1. Lastly, the GSK2394002 was terminated in the preclinical phase after increased arterial pressure and elevated ST was observed in cynomolgus monkey [31]. These ADAMTS-5 inhibitors have all shown to inhibit aggrecan degradation in cartilage explant cultures and/or mouse models [12,32,33]. GSK2384002 and GLPG1972 have shown to decrease ARGS in human OA cartilage explants or OA patients [12,34]. Similar to M6495, GLPG1972 decreased exAGNxI in a dose-dependent manner in human cartilage explants [32]. GSK2384002 decreased the ARGS epitope in human cartilage in the same manner as M6495 in the current study [12]. In the current ex vivo study, we did not find that the tested M6495 doses were toxic to the explants, as the metabolic activity was not affected by M6495, whereas metabolic activity was lowered by O + T treatment at the later time points. Thus, ADAMTS-5 inhibition seems promising as a treatment for OA. In our studies we stimulated the cartilage explant models with pro-inflammatory cytokines, either O + T, IL-1a or O + I, to achieve cartilage degradation by induction of proteolytic activity. A combination of O + T added to bovine cartilage explants have been shown to mimic the cartilage degradation occurring in OA driven by pro-inflammatory factors [35,36]. The pro-inflammatory induction by either IL-1a or O + I have in our labs been shown to induce similar proteolytic activity in cartilage as O + T (unpublished). The enzymatic processes induced by O + T have demonstrated that aggrecanase driven aggrecan degradation happens prior to the induction of MMP-mediated aggrecan and type II collagen degradation [10,37]. This cascade of events has also been observed in mice, where aggrecan degradation preceded collagen degradation and depletion of ADAMTS-5, but not MMP13, halted irreversible cartilage degradation [9,10,11]. The delayed release of MMP-derived cartilage degradation products could be partly explained by the requirement of proteolytic activation of MMPs before they can act as latent zymogens. Thus, the activation of inactive MMPs might be a key step in irreversible cartilage degradation.

In our study, the level of degradation of aggrecan and type II collagen were different between culture types. The huARGS increase in healthy human cartilage explants was similar to OA human cartilage explants, whereas O + T failed to increase exAGNxI and C2M in these healthy human explants. This may indicate that structural differences between the healthy and OA human cartilage alter the proteolytic activity. The difference in aggrecan remodeling assessed by exAGNxI could indicate an easier accessible matrix for the proteases in the OA human cartilage, which in turn leads to increased remodeling of aggrecan and collagen by MMP’s. Considering the differences in matrix turnover and the tissue from OA patients, treatment with M6495 in the OA human cartilage model is arguably the most representative of the single tissue cartilage models from a translational perspective. The fact that M6495 reduces both aggrecan and type II collagen degradation in this model suggests that M6495 may be a good candidate to halt structural changes associated with OA.

The advantage of using the cartilage explant models compared to a chondrocyte model for investigation for the effect of M6495 is that the chondrocytes in the explant models are in the native ECM. As M6495 inhibits active proteases, the major effect is expected on the lowering of tissue destructive mechanisms leading to reductions in degradation markers, while having limited effect on the chondrocyte mediated cartilage formation. In line with this, no effect of M6495 was observed on the type II collagen formation marker exPRO-C2.

The bovine co-culture model, where the synovium is added to induce a pro-inflammatory response, indicated that M6495 inhibited aggrecan deterioration by GAG and type II collagen degradation. The co-culture model system may represent a more representative pro-inflammatory environment compared to the cartilage cultures, where inflammation is induced by adding single cytokines. This might be the reason why C2M release was induced under co-culture conditions but not in bovine explants stimulated with cytokines.

The reduction in GAG observed in the co-culture model underlines that M6495 is halting cartilage degradation. However, this co-culture model system also displayed a higher variance compared to the single cartilage cultures. It is understood that the OA population is not a homogenous population but consists of several subpopulations with specific phenotypes or endotypes, such as trauma induced, inflammatory driven (synovial), and bone or cartilage driven phenotypes [38]. As such, using co-culture model systems with patient-derived synovium (representing different pathogenesis/pathologies) may be the most translational way of inducing cartilage degradation. It is probable that in order to accurately describe the effect of a DMOAD, different models representing pathology of the different phenotypes may be needed. The explant models applied in the current study are driven by an inflammatory component such as added cytokines or synovium. M6495 inhibits the ADAMTS-5, which is downstream of the pro-inflammatory cascade initiated by the cytokines or synovium and thus joint destruction driven by other phenotypes are likely to benefit from a reduction in enzymatic degradation of cartilage.

There are several limitations to this study. Firstly, we tested all aggrecan biomarkers at day five. We know from previous studies that aggrecan degradation is a dynamic process and that assessment by huARGS and exAGNxI may not have the same peak release, thus defining a specific day could potentially mask additional effects at adjacent days. Previous data have shown that exAGNxI peaks at day seven, but was significantly increased already from day three [5]. In the current study the exAGNxI peak differed between the different cultures. In bovine cartilage explants exAGNxI peaked at day five, OA human cartilage explants at day 10 and in healthy human cartilage the exAGNxI level was continuously high from day 7 onwards. This difference is likely also partly attributeble to the state of the cartilage upon initiation of culture. Importantly, despite differences in peak concentration timepoints, an overall decrease in biomarkers in response to M6495 was observed. Secondly, only a single biological replicate was performed on healthy human cartilage, and thus interpretation of the results should be done with caution. Finally, we only assessed type II collagen formation and did not assess aggrecan formation. This could have been tested with CS846 or incorporation of radioactive sulphate.

In conclusion, M6495 is potent anti-catabolic drug which inhibits aggrecan and type II collagen degradation in both bovine and human cartilage explant cultures and bovine synovial co-cultures. These findings indicate that M6495 may be a novel treatment of OA by halting cartilage degradation and preserving knee structure.

## 4. Materials and Methods

### 4.1. Materials

Materials were purchased from Sigma-Aldrich (Darmstadt, Germany) or R&D systems (Abingdon, UK) if not otherwise stated. Cow knees were bought from Harald Hansen Slaughterhouse in Slangerup, Denmark or the slaughterhouse in Brensbach, Germany and human osteoarthritis cartilage and synovial membrane were retrieved from Gentofte Hospital in Gentofte, Denmark or the Elisabethenstift in Darmstadt, Germany within the scope of ethical permissions (H-D-2007-0084 approved in 2007 and FF24/2015 approved in 2015, respectively). Healthy human cartilage was purchased from Asterand Bioscience (Detroit, MI, USA).

### 4.2. Affinity Determination of M6495 for Its Target ADAMTS-5

To determine the affinity of M6495 for ADAMTS-5, the Kinetic Exclusion Assay (KinExA) platform was used. Human ADAMTS-5 was coupled to polymethylmethacrylate (PMMA) beads by incubating 200 mg PMMA beads with 1 mL of a 30 μg/mL ADAMTS-5 solution for 2 h at room temperature while tumbling the vial up and down. After the incubation, the beads were allowed to settle, and the supernatant was discarded. Then, 1 mL of blocking solution (phosphate buffered saline (PBS) + 1% bovine SA (BSA) + 0.02% NaN_3_) was added and the beads were blocked for 1 h at room temperature while tumbling the vial up and down. After the blocking step, the beads were transferred into a glass bead vial and diluted to 30 mL using running buffer (PBS + 0.02% NaN_3_), upon which the beads were ready to use.

A dose response curve (DRC) of ADAMTS-5 was prepared starting at 20 nM with a 1/2.2 dilution factor, and with the last point being a blank (= no ADAMTS-5). This DRC was prepared in sample buffer (PBS + 0.1% BSA + 0.02% NaN_3_) spiked with 20 pM M6495 (final concentration). This mixture was pre-incubated for 24 to 48 h and subsequently injected via KinExA’s auto-sampler over the human ADAMTS-5-conjugated PMMA beads, to capture free Nanobody on the beads. Lastly, captured Nanobody was detected with Alexa Fluor (AF) 647-labeled anti-Nanobody monoclonal antibody (mAb; Ablynx, Ghent, Belgium). Percent free Nanobody was plotted as a function of ADAMTS-5 concentration and fitted using the KinExA Pro Software v. 3.6.5 to determine the KD.

### 4.3. Ligand Binding Assay to Determine the Specificity of M6495

The specificity of M6495 towards ADAMTS-5 was assessed by binding experiments on homologous metalloproteinases which were selected based on a phylogenetic sequence analysis. ADAMTS-1, ADAMTS-4 and ADAMTS-15 were selected and binding of M6495 was assessed using a ligand binding assay. Briefly, ADAMTS-1, ADAMTS-4 or ADAMTS-15 were coated overnight at 1 µg/mL onto 96 well Maxisorp ELISA plates. The plates were blocked with Super Block T20 (PBS). After blocking, a dose-response curve of M6495 (0.0001–1000 nM) or a positive control antibody (to its respective coated target) was applied on the coated plate and incubated for 1 h at room temperature. On the ADAMTS-1 coated plate, bound M6495 Nanobody was detected with an anti-Nanobody mAb (Ablynx), followed by an horseradish peroxidase (HRP) labelled goat anti-mouse-pAb, while the (HIS-tagged) positive control Ab was detected with HRP labelled mouse anti-HIS mAb. On the ADAMTS-4 and ADAMTS-5 coated plates, bound M6495 Nanobody was detected with the biotinylated anti-Nanobody pAb, while the positive control mAbs were detected with a biotinylated goat anti-mouse pAb. For both, streptavidin-HRP (Thermo Scientific, Waltham, MA, USA, No: 21126) was used as a secondary detection tool. All plates were visualized by adding s(HS)TMB (soluble (high sensitivity) 3,3′,5,5′-Tetramethylbenzidine) solution. All dilutions and detection tools were prepared in assay diluent (PBS + 10% Superblock + 0.05% Tween 20). The colouring reaction was stopped after 2.5 min by adding 1M HCl. Optical density was measured at 450 n with 620 nm as reference wavelength.

### 4.4. Ex Vivo Cultures

#### Retrieval of Explants

The full-depth cartilage explants (BEX) extending from the superficial layer to the subchondral bone was isolated by biopsy punchers with a three or four (single cultures) to six mm (co-culture) diameter (Miltex, Yorklyn, PA, USA) as previously described [39]. BEX were harvested from the femoral condyle of the hind leg of cows aged 18–24 months retrieved from the local slaughterhouse maximum 48 h after slaughtering. Human OA cartilage explants (HEX) were harvested from articular cartilage from any part of the joint with visual intact cartilage from OA patients undergoing a total knee replacement or total hip replacement as previously described [40,41,42,43]. Healthy human cartilage (hHEX) was retrieved from a deceased Caucasian male, age 46 years, who died from myocardial infraction. The isolated cartilage explants were randomly distributed in 96-well culture plates and in 48-well plates for co-culture with synovial membrane. The explants were washed three times in sterile PBS, before cultured overnight in DMEM/F12-GlutaMAX for the cultures in Denmark and for the cultures from Germany in DMEM high glucose with 1% Penicillin and Streptomycin (Sigma) ascorbic acid (Sigma, 50 µg/mL) and amphotericin B (Pan Biotech, Aidenbach, Germany), 250 μg/mL) at 37 °C, 5% CO_2_ before experimental start.

Bovine synovial membrane biopsies were collected from the same cow knee as the articular cartilage. Briefly, the synovial tissue was cleaned of visible fat and cut into explants of 30 mg ± 3 mg. The explants were washed three times in sterile PBS, followed by culturing overnight in DMEM high glucose with 1% Penicillin and Streptomycin (Sigma) and ascorbic acid (Sigma, 50 μg/mL) and amphotericin B (Pan Biotech, 250 μg/mL) at 37 °C, 5% CO_2_ before experiment start. Co-cultures of cartilage and synovial membrane were performed in 48-well-plates.

### 4.5. Study Setup

For determination of huARGS and exAGNxI, bovine cartilage was retrieved for single culture from three cows. In the first study, the explants were cultured for three weeks with or without pro-inflammatory stimulation by oncostatin M (10 ng/mL) and tumour-necrosis factor alpha (20 ng/mL) in combination (O + T). M6495 (1, 3, 10, 30, 100, 300 and 1000 nM) was added simultaneously with O + T. Each condition consisted of six replicate explants. The second study was alike the first study, except for the concentration of M6495 tested (0.46, 1.3, 4.11, 12.3, 37, 111, 333 and 1000 nM), which was slightly different and the explants (*n* = 12) were retrieved from two cows (six explants per cow). A control condition receiving Insulin-like Growth factor 1 (IGF-1) (100 ng/mL) was included in the first study and as a positive control for cartilage formation in the second study. For determination of GAG (glycosaminoglycans), bovine explants from another cow were treated with the pro-inflammatory cytokine IL-1α (10 ng/mL) with or without M6495 (3, 10, 30, 100, 300, 1000, 3000, 10,000 nM) for 5 days. Human cartilage explants were treated with IL-1β (10 ng/mL) + oncostatin M (50 µg/mL) with or without M6495 (3, 10, 30, 100, 300, 1000, 3000, 10,000 nM). Treatment started directly at the day of arrival.

To determine huARGS and exAGNxI in human explants (HEX) for single culture, cartilage was retrieved from eight patients. The explants were cultured for two weeks with or without O + T stimulation. M6495 (0.46, 1.3, 4.11, 12.3, 37, 111, 333 and 1000 nM) was added simultaneously with O + T. Each condition consisted of one to four replicate explants from each patient with a total of 21 explants. hHEX was cultured for three weeks with or without O + T stimulation. M6495 (2, 10 and 50 nM) was added simultaneously with O + T. Each condition consisted of six replicate explants.

Bovine co-culture (bCC) consisted of cartilage and synovium from one cow. The explants were cultured for five weeks without further stimulation. M6495 (1, 10, 100 nM) was added at experimental start. All conditions consisted of four individual explants.

For all explant studies, conditioned media were collected three times per week. For the co-culture study, supernatant was collected weekly. At the same time, freshly prepared medium with treatments was added. Conditioned medium was stored at −20 °C for further analyses.

### 4.6. Biomarkers

#### 4.6.1. Metabolic Activity

Metabolic activity was measured at baseline (day 0) and at termination. The non-toxic AlamarBlue Reagent (Life Technologies, Carlsbad, CA, USA) was used to quantify the explants metabolic activity following manufactures instructions with incubation for one (synovial membrane) or three hours (cartilage) The readout was corrected for background by subtracting the average of four controls wells from the result of each other well.

#### 4.6.2. ECM Turnover

Collagen type II turnover was quantified by neo-epitope biomarkers assessing degradation and formation. MMP-degraded type II collagen (C2M) is a validated ELISA [40] detecting a neo-epitope located in the helical region of type II collagen. Formation of type II collagen was quantified by the PRO-C2 ELISA [43] detecting the N-terminal pro-peptide of type II pro-collagen. GAG release in the supernatant was determined with the dimethyl methylene blue (DMMB) method as previously described [44].

Aggrecan degradation was quantified by two neo-epitope biomarkers assessing the well-known aggrecanase derived degradation site in the IGD of aggrecan at amino acid Glu373-374Ala. HuARGS quantifies the N-terminal cleavage fragment, ARGSVI, and the previously described exAGNxI [45] quantifies the C-terminal cleavage site, NITEGE. The technically validated huARGS ELISA applied the OA-1 antibody (GSK, Philadelphia, PA, USA) with an in-house antibody of Nordic Bioscience (Herlev, Denmark). The intra- and inter-variation of the huARGS ELISA were acceptable (<10% and <15% respectively), the accuracy was accepted (<15%), dilution recovery was acceptable (<20%) and no interference of lipids, biotin and human anti-mouse antibodies (HAMA) was observed.

### 4.7. Statistics

The BEX data presented of huARGS and exAGNxI originated from the second study and was presented as percent of O + T stimulation calculated individually per cow (*n* = 2). All other studies were reported as μg/mL, ng/mL or nM, depending on the biomarker. Reported values were mean values and standard error of mean (SEM) if not otherwise stated. Statistical differences on individual days were tested by one-way analysis of variance (ANOVA) with Dunnett’s multiple comparisons test comparing to O + T assuming normal distribution. Statistical significance over time were done by two-way repeated measures ANOVA with Dunnett’s multiple comparisons test comparing to O + T assuming normal distribution. Graphical illustration and statistical tests were performed in GraphPad Prism v. 7.00 for Windows, GraphPad Software, La Jolla, CA, USA, www.graphpad.com. Statistical significance was considered when * *p* < 0.05, ** *p* < 0.01 and *** *p* < 0.001.

## Supplementary Materials

Supplementary materials can be found at https://www.mdpi.com/1422-0067/21/17/5992/s1. Figure S1: M6495 binds Variant B and Variant C variant with similar affinity, but does not bind to the Variant A.; Figure S2: Metabolic activity of cartilage explants during the study period; Figure S3: Aggrecan and type II collagen degradation in bovine cartilage explants study 1. The aggrecan biomarkers were assessed in the initial period of the study culture, whereas type II collagen was assessed in the last period of the culture; Figure S4: Aggrecan and type II collagen degradation in bovine cartilage explants study 2. huARGS was assessed at day 5 of study culture. exAGNxI was assessed during the first 14 days of culture. Type II collagen turnover was assessed from day 7 to 14 during the studies; Figure S5: Results of the co-culture study throughout the study. Aggrecan turnover was assessed by GAG and type II collagen degradation by C2M.

## Abbreviations

AGNxI	Aggrecanase degraded aggrecan (NITEGE)
bCC	Bovine co-culture of cartilage and synovial membrane
BEX	Bovine cartilage explant model
bSMEx	Bovine synovial membrane explants
C2M	MMP-mediated metabolite of type II collagen
GAG	Glycosaminoglycan
HAMA	Human anti-mouse antibodies
hCC	Human co-culture of cartilage and synovial membrane
HEX	Human OA cartilage explant model
hHEX	Human healthy cartilage explant model
hSMEx	Human synovial membrane explants
huARGS	Aggrecanase degraded aggrecan (ARGSVI)
HRP	Horseradish peroxidase
IGD	Interglobular domain
IGF-1	Insulin-like Growth factor 1
M6495	Nanobody targeting ADAMTS-5
mAb	Monoclonal antibody
O + T	OSM together with TNF-a
OSM	Oncostatin M
PRO-C2	Formation metabolite of type II collagen
s(HS)TMB	Soluble (high sensitivity) 3,3′,5,5′-Tetramethylbenzidine
TNF-a	Tumor necrosis factor alpha
*w*/*o*	Without treatment
