# Supplementary material for: The Anti-ADAMTS-5 Nanobody® M6495 Protects Cartilage Degradation Ex Vivo
Source: Int J Mol Sci. 2020 Aug 20;21(17):5992. doi: 10.3390/ijms21175992 (PMC7503673; doi:10.3390/ijms21175992)
Supplement: Supplementary file 1 [file ijms-21-05992-s001.pdf]

International Journal of
Molecular Sciences

MDPI

Supplementary Materials

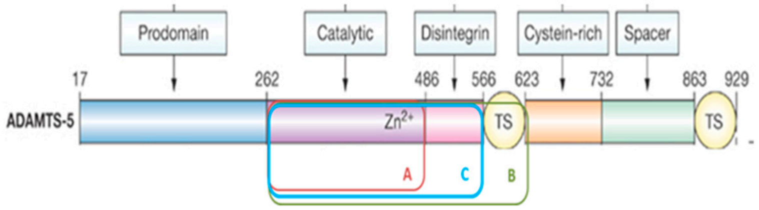

Figure S1. M6495 binds Variant B and Variant C variant with similar affinity, but does not bind to the Variant A.

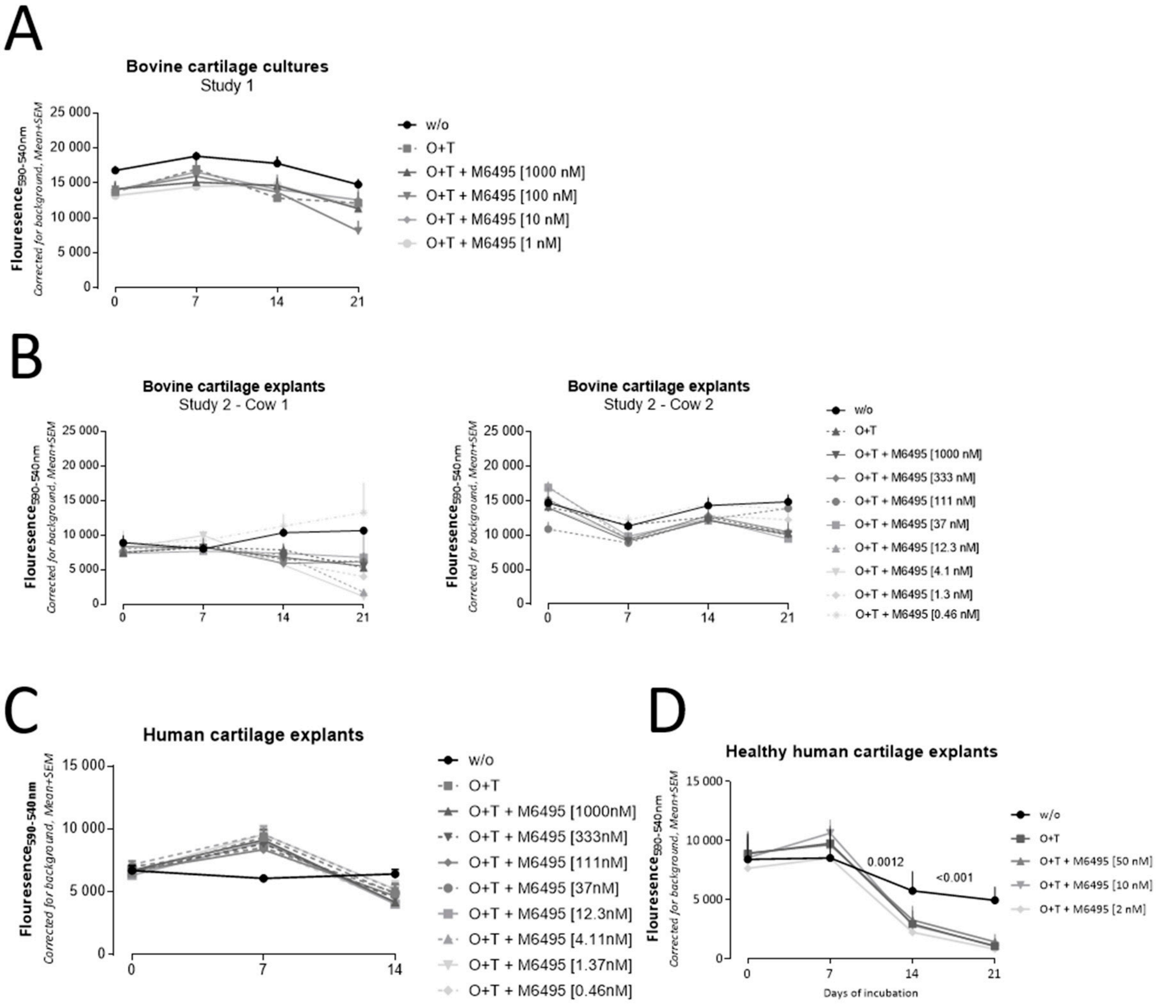

Figure S2. Metabolic activity of cartilage explants during the study period. (**A**): Bovine Cartilage culture study 1. (**B**): Bovine cartilage culture study 2. (**C**): Human osteoarthritic cartilage. (**D**): Healthy human cartilage. Statistical significance was tested by repeated measure Two-way ANOVA with Dunnett's multiple comparisons test comparing to O + T or baseline.

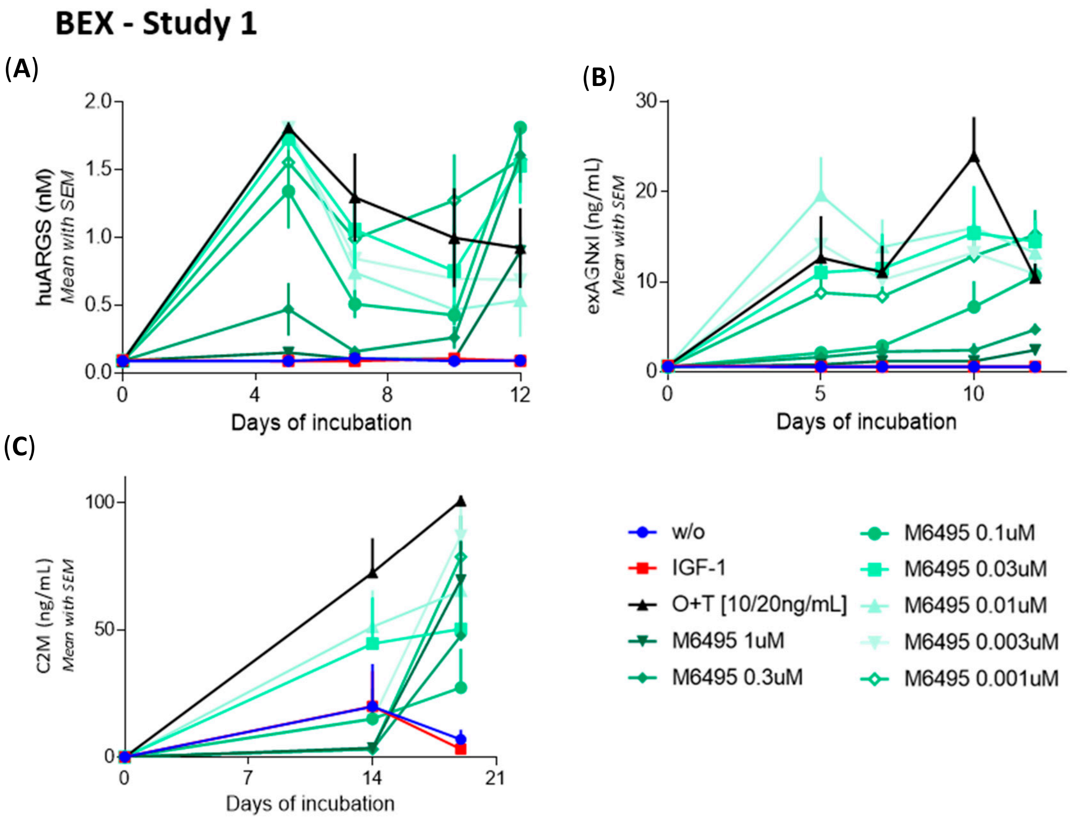

Figure S3. Aggrecan and type II collagen degradation in bovine cartilage explants study 1. The aggrecan biomarkers were assessed in the initial period of the study culture, whereas type II collagen was assessed in the last period of the culture. (**A**): ADAMTS-5 derived aggrecan degradation (huARGS). (**B**): MMP-derived aggrecan degradation (exAGNxI). (**C**): MMP-derived collagen type II degradation (C2M). Statistical significance was tested by repeated measure Two-way ANOVA with Dunnett's multiple comparisons test comparing to O + T or baseline.

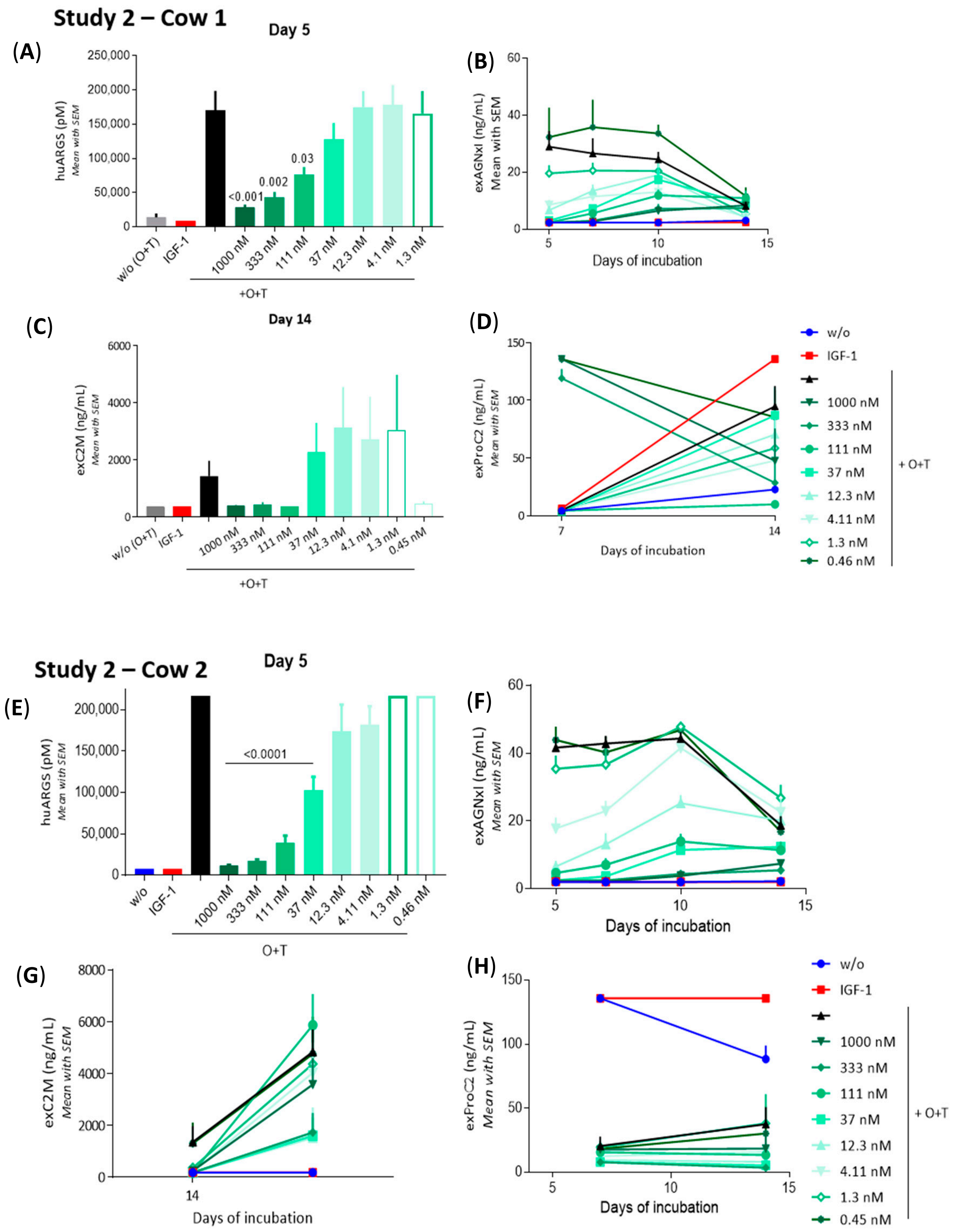

Figure S4. Aggrecan and type II collagen degradation in bovine cartilage explants study 2. huARGS was assessed at day 5 of study culture. exAGNxI was assessed during the first 14 days of culture. Type II collagen turnover was assessed from day 7 to 14 during the studies. (**A**,**E**): ADAMTS-5 derived aggrecan degradation huARGS) in study 1 and 2, respectively. (**B**,**F**): MMP-derived aggrecan degradation in study 1 and 2, respectively. (**C**,**G**): MMP-derived collagen type II degradation (exC2M) in study 1 and 2, respectively. (**D**,**H**): Formation of type II collagen (exPRO-C2) in study 1 and 2, respectively. Statistical significance was tested by repeated measure Two-way ANOVA with Dunnett's multiple comparisons test comparing to O + T or baseline or by one-way ANOVA with Dunnett's multiple comparisons test comparing to O + T

(**A**)

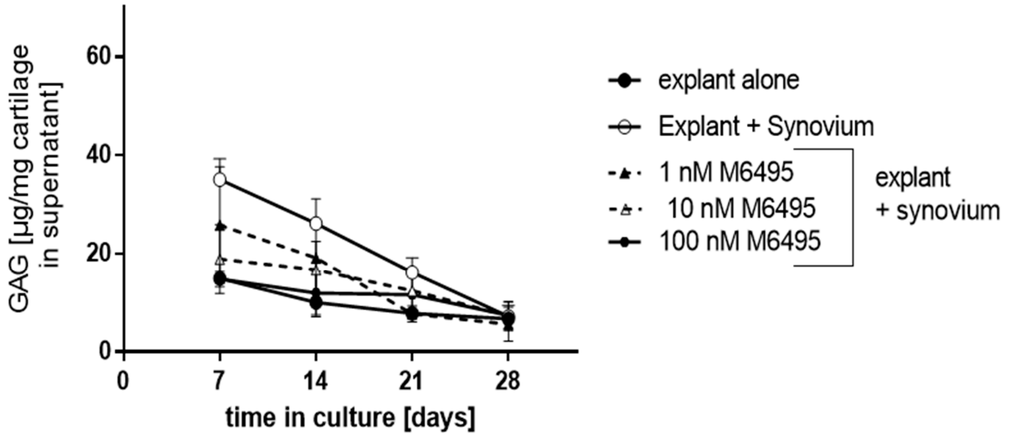

(**B**)

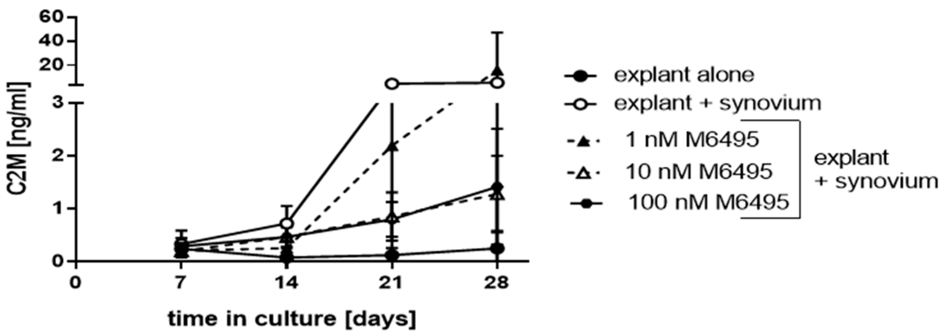

Figure S5. Results of the co-culture study throughout the study. Aggrecan turnover was assessed by glycosaminoglycan (GAG) (**A**) and MMP-derived type II collagen degradation by C2M (**B**). Statistical significance was tested by repeated measure Two-way ANOVA with Dunnett's multiple comparisons test comparing to explant + synovium or day 7.
